# *ompW* is cooperatively upregulated by MarA and SoxS in response to menadione

**DOI:** 10.1099/mic.0.066050-0

**Published:** 2013-04

**Authors:** B. Collao, E. H. Morales, F. Gil, I. L. Calderón, C. P. Saavedra

**Affiliations:** Laboratorio de Microbiología Molecular, Facultad de Ciencias Biológicas, Universidad Andres Bello, Santiago, Chile

## Abstract

OmpW is a minor porin whose biological function has not been clearly defined. Evidence obtained in our laboratory indicates that in *Salmonella enterica* serovar Typhimurium the expression of OmpW is activated by SoxS upon exposure to paraquat and it is required for resistance. SoxS belongs to the AraC family of transcriptional regulators, like MarA and Rob. Due to their high structural similarity, the genes under their control have been grouped in the *mar*/*sox*/*rob* regulon, which presents a DNA-binding consensus sequence denominated the *marsox* box. In this work, we evaluated the role of the transcription factors MarA, SoxS and Rob of *S. enterica* serovar Typhimurium** in regulating *ompW* expression in response to menadione. We determined the transcript and protein levels of OmpW in different genetic backgrounds; in the wild-type and Δ*rob* strains *ompW* was upregulated in response to menadione, while in the Δ*marA* and Δ*soxS* strains the induction was abolished. In a double *marA soxS* mutant, *ompW* transcript levels were lowered after exposure to menadione, and only complementation *in trans* with both genes restored the positive regulation. Using transcriptional fusions and electrophoretic mobility shift assays with mutant versions of the promoter region we demonstrated that two of the predicted sites were functional. Additionally, we demonstrated that MarA increases the affinity of SoxS for the *ompW* promoter region. In conclusion, our study shows that *ompW* is upregulated in response to menadione in a cooperative manner by MarA and SoxS through a direct interaction with the promoter region.

## Introduction

Porins are aqueous channels that allow the passive diffusion of hydrophilic solutes, nutrients or toxic compounds through the bacterial outer membrane and participate, at least in part, in the ability of bacteria to adapt to diverse environments, in drug resistance mechanisms and in bacterial pathogenesis (Benz & Bauer, 1988; Chatfield *et al.*, 1991; Jeanteur *et al.*, 1991; Weiss *et al.*, 1991; Groisman & Ochman, 1994; Nikaido, 1996; Zgurskaya & Nikaido, 2000; Koebnik *et al.*, 2000; Rodríguez-Morales *et al.*, 2006). Some years ago, Morimyo (1988) isolated and characterized *Escherichia coli* mutants sensitive to paraquat, a superoxide-generating compound (Hassan & Fridovich, 1979). The deleted region in *E. coli* is highly conserved in *Salmonella enterica* serovar Typhimurium and contains the *ompW* gene (Gil *et al.*, 2007), which encodes a minor porin that has been well studied and its structure in *E. coli* and *Vibrio cholerae* has been described, although its biological function has not been clearly defined (Nandi *et al.*, 2005; Hong *et al.*, 2006). It is thought to be involved in osmoregulation, since in *Vibrio alginolyticus* high salt concentrations (NaCl 4 %) induce its expression (Xu *et al.*, 2005). Furthermore, a *S. enterica* serovar Typhimurium** ceftriaxone-resistant strain showed decreased expression of *ompW*, suggesting that it might be involved in the uptake of this antibiotic (Hu *et al.*, 2005). Evidence obtained in our laboratory indicates that in *S. enterica* serovar Typhimurium 14028s OmpW expression is increased in the presence of paraquat and it mediates resistance (Gil *et al.*, 2007).

The cellular response to superoxide (O_2_^−^) is regulated at the transcriptional level by the SoxRS system (Greenberg *et al.*, 1990). Upon exposure to O_2_^−^ and/or ammonium quaternary compounds, SoxR is oxidized and converted to an active form that induces the transcription of *soxS*, which binds to the promoter regions of several genes whose products are involved in the response to oxidative damage (Storz & Imlay, 1999; Scandalios, 2002; Imlay, 2008; Gu & Imlay, 2011). In this context, the evidence supports a model in which ammonium quaternary compounds are responsible for SoxR activation (Krapp *et al.*, 2011; Gu & Imlay, 2011); however, it has been recently confirmed that O_2_^−^ is also able to oxidize its 2Fe–2S cluster (Fujikawa *et al.*, 2012). SoxS belongs to the AraC family of transcriptional regulators, of which MarA and Rob are also members (Martin & Rosner, 2002). In *E. coli* MarA, SoxS and the N-terminal domain of Rob, which include the DNA-binding domain, share approximately 50 % amino acid sequence identity (Jair *et al.*, 1995, 1996; Tobes & Ramos, 2002). The *mar*/*sox*/*rob* regulons overlap and together they co-regulate, by direct binding to the promoter regions, more than 40 different genes (Aono *et al.*, 1998; Martin *et al.*, 1999, 2000; Martin & Rosner, 2002). Expression of MarA, SoxS and Rob is increased upon exposure to a wide variety of signals. MarA is increased in response to weak acid conditions and salicylate treatment (Pomposiello *et al.*, 2001); SoxS in response to nitric oxide, superoxide and ammonium quaternary compounds (Li & Demple, 1994; Vasil’eva *et al.*, 2001); and Rob after treatment with bile salts and dipyridyl (Storz & Imlay, 1999; Semchyshyn *et al.*, 2005; Kwon *et al.*, 2000). In *E*. *coli*, their upregulation is correlated with changes in the expression of genes involved in the efflux of antibiotics (*acrAB* and *tolC*), decrease in outer-membrane permeability (*micF*), superoxide resistance (*fpr* and *sodA*), DNA repair systems (*nfo*) and those with unknown function (Aono *et al.*, 1998; Pomposiello *et al.*, 2001; Chollet *et al.*, 2002, 2004; Giró *et al.*, 2006; Lee *et al.*, 2009). Due to the high structural similarity among these proteins, genes under their control have been denominated the *mar*/*sox*/*rob* regulon, which present a DNA-binding consensus sequence at their promoter regions, denominated the *marsox* box which is degenerate and asymmetrical (AYnGCACnnWnnRYYAAAY), and has been detected at various locations on the chromosome of *E*. *coli* (Martin *et al.*, 2008; Aono *et al.*, 1998; Martin *et al.*, 1999, 2000; Martin & Rosner, 2002). These binding sites are also configured in a specific orientation and relative distance to the upstream −35 and −10 elements, to which RNA polymerase binds (Martin & Rosner, 2002; Selke *et al.*, 2007).

Previous work in our laboratory determined that *ompW* is regulated by SoxS in response to paraquat, and a *marsox* box has been defined at its promoter region with the sequence 5′-TTTGCATAGCGTGAATATGTCAAAATTGAT-3′ (Gil *et al.*, 2009). Since the binding sites of the members of the *mar*/*sox*/*rob* regulon are similar, in *S. enterica* serovar Typhimurium** Rob and MarA might also regulate *ompW* in response to menadione, another superoxide-generating compound (Kato *et al.*, 1994).

In the present work, we evaluated the effect of menadione on *ompW* expression and the role of MarA and SoxS in the response. To evaluate the changes after exposure to menadione, we determined the transcript and protein levels of OmpW in the different genetic backgrounds after exposure to the toxic compound. In the wild-type and Δ*rob* strains, *ompW* was upregulated in response to menadione, while deletion of MarA or SoxS abolished the regulation. Bioinformatic analyses predicted the presence of three potential *marsox* boxes at the *ompW* promoter region, including the one previously described by Gil *et al.* (2009). Using transcriptional fusions and electrophoretic mobility shift assays (EMSAs) with the wild-type and mutated promoter regions we demonstrated that two of the predicted sites were functional. Interestingly, in a double *marA soxS* mutant strain *ompW* transcript levels were lowered after menadione exposure, and only complementation *in trans* with both genes was able to restore the positive regulation observed in the wild-type strain. In conclusion, we demonstrated that in response to menadione, MarA and SoxS cooperatively regulate *ompW* through a direct interaction with the promoter region.

## Methods

### 

#### Bacterial strains and growth conditions.

*Salmonella* strains used in this study are listed in Table 1. Bacteria were grown routinely at 37 °C in Luria–Bertani (LB) broth with shaking. When required, LB was supplemented with ampicillin (100 mg l^−1^) or kanamycin (50 mg l^−1^). Solid medium included 15 g agar l^−1^. When necessary, growth medium was treated with menadione (50 µM).

**Table 1.** Bacterial strains used in this study

Strain	Relevant characteristic(s)	Source or reference
***S. enterica* serovar Typhimurium**		
14028s	Wild-type	G. Mora, Universidad Andres Bello, Chile
Δ*soxS*	*soxS* : : Cam	Gil *et al.* (2009)
Δ*soxS*/pBAR322-*soxS*	Δ*soxS* strain complemented with pBR322 vector carrying the *S. enterica* serovar Typhimurium *soxS* gene and its promoter	This work
Δ*soxS*/pBR322	Δ*soxS* strain with empty pBR322 vector	This work
Δ*marA*	*marA* : : Kan	Collao *et al.* (2012)
Δ*marA*/pBR322-*marA*	Δ*marA* strain complemented with pBR322 vector carrying the *S. enterica* serovar Typhimurium *marA* gene and its promoter	This work
Δ*marA*/pBR322	Δ*marA* strain with empty pBR322 vector	This work
Δ*rob*	*rob* : : Cam	Collao *et al.* (2012)
Δ*rob*/pBR322-*rob*	Δ*rob* strain complemented with pBR322vector carrying the *S. enterica* serovar Typhimurium *rob* gene and its promoter	This work
Δ*rob*/pBR322	Δ*rob* strain with empty pBR322 vector	This work
*ompW*-3×-FLAG	Strain carrying the epitope-tagged *ompW* gene	Gil *et al.* (2009)
Δ*marA ompW*-3×-FLAG	*marA* mutant strain carrying the epitope-tagged *ompW* gene	This work
Δ*soxS ompW*-3×-FLAG	*soxS* mutant strain carrying the epitope-tagged *ompW* gene	This work
Δ*rob ompW*-3×-FLAG	*rob* mutant strain carrying the epitope-tagged *ompW* gene	This work
Δ*marA* Δ*soxS*	*marA* : : Kan *soxS* : : Cam	Collao *et al.* (2012)
Δ*marA* Δ*soxS*/pB322-*soxS*	Δ*marA* Δ*soxS* strain complemented with pBR322 vector carrying the *S. enterica* serovar *Typhimurium* *soxS* gene	This work
Δ*marA* Δ*soxS*/pB322-*marA*	Δ*marA* Δ*soxS* strain complemented with pBR322 vector carrying the *S. enterica* serovar *Typhimurium* *marA* gene	This work
Δ*marA* Δ*soxS*/pB322-*marA-soxS*	Δ*marA* Δ*soxS* strain complemented with pBR322 vector carrying the *S. enterica* serovar *Typhimurium* *marA* and *soxS* genes	This work
14028s/p*ompW*-*lacZ*	Wild-type strain with pLacZ vector carrying *ompW* promoter	This work
14028s/pMutA-*lacZ*	Wild-type strain with pLacZ vector carrying *ompW* promoter with MS-A mutant	This work
14028s/pMutB-*lacZ*	Wild-type strain with pLacZ vector carrying *ompW* promoter with MS-B mutant	This work
14028s/pMutC-*lacZ*	Wild-type strain with pLacZ vector carrying *ompW* promoter with MS-C mutant	This work
14028s/pMutAB-*lacZ*	Wild-type strain with pLacZ vector carrying *ompW* promoter with MS-A and MS-B mutants	This work
14028s/pMutAC-*lacZ*	Wild-type strain with pLacZ vector carrying *ompW* promoter with MS-A and MS-C mutants	This work
14028s/pMutBC-*lacZ*	Wild-type strain with pLacZ vector carrying *ompW* promoter with MS-B and MS-C mutants	This work
14028s/pMutABC-*lacZ*	Wild-type strain with pLacZ vector carrying *ompW* promoter with MS-A, MS-B and MS-C mutants	This work
***E. coli***		
Top10	F^−^ *mcrA* Δ(*mrr*-*hsdRMS*-*mcrBC*) ϕ80*lacZ*ΔM15 Δl*acX74 nupG recA1 araD139* Δ(*ara-leu*)7697 *galE*15 *galK*16 *rpsL*(Str^R^) *endA1* λ^−^	Invitrogen
BL21(DE3)	F^−^ *ompT gal dcm lon hsdS*_B_() λ(DE3 [*lacI lac*UV5-T7 gene 1 *ind1 sam7 nin5*])	Invitrogen
Top10/pET-*marA*	Top10 transformed with the pET-TOPO101 MarA vector carrying the *S. enterica* serovar *Typhimurium* *marA* gene	Collao *et al.* (2012)
BL21(DE3)/pET-*marA*	BL21(DE3) transformed with the pET-TOPO101 MarA vector carrying the *S. enterica* serovar *Typhimurium* *marA* gene	Collao *et al.* (2012)
Top10/pET-*soxS*	Top10 transformed with the pET-TOPO101 SoxS vector carrying the *S. enterica* serovar *Typhimurium* *soxS* gene	Collao *et al.* (2012)
BL21(DE3)/pET-*soxS*	BL21(DE3) transformed with the pET-TOPO101 SoxS vector carrying the *S. enterica* serovar *Typhimurium* *soxS* gene	Collao *et al.* (2012)

#### Bioinformatic analysis.

Bioinformatic analyses in search for *marsox* boxes at the *ompW* promoter region were performed using the Vector NTI software using the sequences described by Martin *et al.* (1999) and Gil *et al.* (2009).

#### Construction and cloning of strains.

For the construction of the double mutant strains we used bacteriophage P22 HT105/1 *int* −201 using one single-mutant strain as the donor and the other as the recipient (Ebel-Tsipis *et al.*, 1972). The presence of substitution mutations was confirmed by PCR using specific primers (Table 2).

**Table 2.** Primers used in this study Underlined sequences indicate restriction sites for *Kpn*I or *Hin*dIII which were introduced in the primers. Sequences in bold type indicate restriction sites for *Eco*RI or *Bam*HI introduced in the primers. Sequences in italics represent complementary sequences added to generate overlapping PCR products to produce the divergent *marA*-*soxS* construct as described in Methods.

Primer name	Sequence
soxS_Ext_Fw	5′-GAACAGGTTAGCTGGTTGCT-3′
soxS_Ext_Rv	5′-GATTTTTTTTCCATAAATCG-3′
marA_Ext_Fw	5′-GTAGTTGCCATGGTTCAGCG-3′
marA_Ext_Rv	5′-TTGAGTATTTGCTCAAGAAA-3′
rob_Ext_Fw	5′-ACCTGTCACGTTGCCTAAAA-3′
rob_Ext_Rv	5′-GGGTGGTAGAAACCGCAGGG-3′
pOmpW_+1_Fw	5′-AGCAATACCAATATTTTCGCC-3′
pOmpW_+130_Rv	5′-CCGGACTGCACGCATAAAG-3′
pLacZ_OmpW_−600Fw	5′-CGGGGTACCCCCGATATCGAAAATTCGCG-3′
pLacZ_OmpW_+1Rv	5′-CCCAAGCTTACCCGCTCCATCGTTATGGT-3′
pOmpW_MUTA_Fw	5′-GCCTTTATCGCCAGG**AAA**ACAGGAGCAGACAAATATTTGC-3′
pOmpW_MUTA_Rv	5′-GCAAATATTTGTCTGCTCCTGT**TTT**CCTGGCGATAAAGGC-3′
pOmpW_MUTB_Fw	5′-TCGCCAGGGCAACAGGA**AAA**GACAAATATTTGCATAGCGT-3′
pOmpW_MUTB_Rv	5′-ACGCTATGCAAATATTTGTCT**TTT**CCTGTTGCCCTGGCGA-3′
pOmpW_MUTC_Fw	5′-GGAGCAGACAAATATTT**AAA**TAGCGTGAATATGTCAAAAT-3′
pOmpW_MUTC_Rv	5′-ATTTTGACATATTCACGCTA**TTT**AAATATTTGTCTGCTCC-3′
pBR322_MarAR_EcoRI_Fw	5′-**CCGGAATTC**CTAGTAGTTGCCATGGTTCA-3′
pBR322_MarAR_complsoxS_Rv	5′- CCGCCGCGAGTTCGATCGCA*CTCCCAGCGATTACCGTCAAGAAACAGCGCCACGGTGGTT*-3′
pBR322_SoxS_complmarA_Fw	5′-CTCCCGTTAGCCAATCCGCT*AACCACCGTGGCGCTGTTTCTTGACGGTAATCGCTGGGAG*-3′
pBR322_SoxS_BamHI_Rv	5′-**CGCGGATCC**TTAATCATCTTCAAGCAGCC-3′
pBR322_MarA_BamHI_Rv	5′-**CGCGGATCC**GAAACAGCGCCACGGTGGTT-3′′
pBR322_SoxS_EcoRI_Fw	5′-**CCGGAATTC**TTGACGGTAATCGCTGGGAG-3′
pBR322_Rob_BamHI_Fw	5′-**CGCGGATCC**GCCCGTTTTCGCCCGGCTAA-3′
pBR322_Rob_EcoRI_Rv	5′-**CCGGAATTC**AAAATATCCCCATCCTTTCA-3′
ompW_RT_Fw	5′-ATGAAAAAATTTACAGTGG-3′
ompW_RT_Rv	5′-GAAACGATAGCCTGCCGA-3′
marA_RT_Fw	5′-TTCATAGCATTTTGGACTGG-3′
marA_RT_Rv	5′-TAGAGAATGGGCTCGTTGCT-3′
soxS_RT_Fw	5′-GCGGATGTTTCGTACGGTAA-3′
soxS_RT_Rv	5′-GGTGACGGTAATCGCTGGGA-3′
rob_RT_Fw	5′-CCGCTGTCACTTGACAATGT-3′
rob_RT_Rv	5′-GTTTGCTGAGAATCGAAGCG-3′
16S_RT_Fw	5′-GTAGAATTCCAGGTGTAGCG-3′
16S_RT_Rv	5′-TTATCACTGGCAGTCTCCTT-3′

Genetic complementation of the Δ*soxS*, Δ*marA*, Δ*rob* and Δ*marA soxS* strains was performed using plasmids pBR322-*soxS*, pBR322-*marA*, pBR322-*rob* and pBR322-*marA*-*soxS*, respectively. To generate these plasmids, *S. enterica* serovar Typhimurium *soxS, marA* and *rob* genes were amplified by PCR using primers listed in Table 2. The PCR was performed under the following conditions: 10 min at 95 °C, followed by 30 cycles of 30 s at 95 °C, 45 s at 55 °C and 1 min at 72 °C, and a final extension of 10 min at 72 °C. The restriction sites (*Eco*RI and *Bam*HI) at the ends of the DNA fragment were introduced in the PCR primers (sequences in bold type in Table 2) and were digested with the corresponding enzymes. The digested PCR product was cloned into the multiple cloning site (MCS) of pBR322. To generate plasmid pBR322-*marA–soxS* primers pBR322_MarAR_EcoRI_Fw with pBR322_MarAR_complsoxS_Rv and pBR322_SoxS_complmarA_Fw with pBR322_SoxS_BamHI_Rv (Table 2) were used to generate overlapping PCR products spanning the divergent construct *marA*–*soxS*, taking advantage of the complementary sequence added (sequences in italic type in Table 2). The resulting PCR products were used as templates in a second reaction with primers pBR322_MarAR_EcoRI_Fw and pBR322_SoxS_BamHI_Rv to generate the divergent construct, which was digested and cloned into the MCS of plasmid pBR322.

#### RNA isolation and mRNA detection.

An overnight bacterial culture was diluted 100-fold with fresh LB medium and was grown at 37 °C with shaking to OD_600_ ~0.4. The culture was split into two 10 ml aliquots and one of them was incubated with 50 µM menadione. Cells were grown at 37 °C and 4 ml aliquots were withdrawn 20 min after menadione exposure. Total RNA was extracted using the GenElute Total RNA purification kit (Sigma) following the manufacturer’s instructions. Total RNA was treated with 2 U DNase I to remove trace amounts of DNA. cDNA synthesis was carried out at 37 °C for 1 h in 25 µl of a mixture that contained 2.5 pmol of the specific primers, 10 µl template RNA (5 µg), 0.2 mM dNTPs, 1 µl sterile water, 4 µl 5× buffer [250 mM Tris/HCl pH 8.3, 375 mM KCl, 15 mM MgCl_2_, 10 mM DTT, 40 U RNasin and 200 U MMLV reverse transcriptase (Invitrogen)]. Relative quantification of the transcript levels of *ompW*, *marA*, *rob* and *soxS* by real-time RT-PCR (qRT-PCR) was performed using the Brilliant II SYBR Green QPCR Master Reagent kit and the Mx3000P detection system (Stratagene). 16S rRNA levels were used for normalization. The qRT-PCR mixture (20 µl) contained 1 µl cDNA template and 120 nM of each primer [ompW_RT_Fw and ompW_RT_Rv for the *ompW* gene, marA_RT_Fw and marA_RT_Rv for the *marA* gene, soxS_RT_Fw and soxS_RT_Rv for the *soxS* gene, rob_RT_Fw and rob_RT_Rv for the *rob* gene, and 16S_RT_Fw and 16S_RT_Rv for the 16S rRNA gene (16S) (Table 2)]. The qRT-PCR was performed under the following conditions: 10 min at 95 °C, followed by 40 cycles of 30 s at 95 °C, 45 s at 53 °C and 30 s at 72 °C, followed by a melting cycle from 53 to 95 °C to check for amplification specificity. A standard quantification curve with serial dilutions of RT-PCR products was constructed for each gene to calculate the amplification efficiency. These values were used to obtain the ratio between the gene of interest and the expression of the 16S rRNA gene as described by Pfaffl (2001). All experiments were performed in three biological and technical replicates.

#### Protein purification.

Briefly, *E. coli* BL21 cells harbouring plasmid pET-TOPO-*soxS* or *marA* were grown in 500 ml LB medium supplemented with ampicillin (100 µg ml^−1^) to OD_600_ ~0.4 and protein overexpression was carried out by adding 1 mM IPTG with further growth for 6 h. His-tagged SoxS and MarA used in EMSAs were purified as previously described (Collao *et al.*, 2012).

#### Immunoblot analysis.

Immunoblotting using an anti-FLAG M2 mAb (Sigma) detected a 3×FLAG-containing fusion protein. Strains carrying the epitope-tagged construct were grown at 37 °C with shaking to OD_600_ ~0.4. The culture was split into two 10 ml aliquots, one of which was incubated with 50 µM menadione. Cultures were grown at 37 °C and after 20 min of exposure cells were centrifuged at 10 000 ***g*** for 3 min. Bacterial pellets were suspended in 100 mM Tris/HCl (pH 8.0) and subjected to three rounds of sonication of 30 s each. After centrifuging at 13 000 ***g*** for 5 min, the pelleted material was subjected to SDS-PAGE and size-separated proteins were electroblotted onto nitrocellulose membranes, incubated with anti-FLAG antibody M2 (1 : 1000 dilution) upon which the FLAG epitope was detected with peroxidase-conjugated anti-mouse IgG and peroxidase activity.

#### Construction of transcriptional fusions with the reporter gene *lacZ*.

The native *ompW* promoter region from positions +1 to −600 (with respect to the translation start site) was amplified by PCR with primers pLacZ_OmpW_−600_Fw and pLacZ_OmpW_+1_Rv using genomic DNA from *S. enterica* serovar** Typhimurium as a template (strain 14028s). The restriction sites (*Kpn*I and *Hin*dIII, respectively) at the ends of the DNA fragment were introduced by the PCR primers (underlined sequences in Table 2) and were digested with the corresponding enzymes. The digested PCR product was cloned into the MCS of the β-galactosidase reporter vector pLacZ-Basic (GenBank accession no. U13184) (Clontech), generating plasmid p*ompW*-*lacZ.* To generate plasmids pMutA-*lacZ*, pMutB-*lacZ* and pMutC-*lacZ*, primers ompW_pLacZ_-600Fw with pOmpW_MUTA_Rv, pOmpW_MUTB_Rv or pOmpW_MUTC_Rv and ompW_pLacZ_+1_Rv with pOmpW_MUTA_Fw, pOmpW_MUTB_Fw or pOmpW_MUTC_Fw (Table 2) were used to generate overlapping PCR products spanning the whole length of the *ompW* promoter. The PCR was performed under the following conditions: 5 min at 95 °C, followed by 10 cycles of 30 s at 94 °C, 30 s at 40 °C and 2 min at 72 °C, followed by 10 cycles of 30 s at 94 °C, 30 s at 45 °C and 2 min at 72 °C, and 20 cycles of 30 s at 94 °C, 30 s at 50 °C and 2 min at 72 °C, and a final extension of 10 min at 72 °C. The resulting PCR products were used as templates in a second reaction with primers pLacZ_OmpW_−600Fw and pLacZ_OmpW_+1Rv to generate the mutated *ompW* promoter, which was digested and cloned into the MCS of plasmid pLacZ-Basic. PCR conditions were 10 min at 95 °C, followed by 30 cycles of 30 s at 95 °C, 30 s at 55 °C and 1 min at 72 °C, and a final extension of 10 min at 72 °C. Mutation of other sites was generated the same way, generating plasmids pMutAB-*lacZ*, pMutAC-*lacZ*, pMutBC-*lacZ* and pMutABC-*lacZ*. Constructions were confirmed by DNA sequencing. The constructs were transformed into strain 14028s. To evaluate activity, cells at OD_600_ ~0.4 were grown for 20 min in the presence of 50 µM menadione. Control cells received no treatment. β-Galactosidase activity was determined as previously described by Gil *et al.* (2007).

#### EMSA.

To study protein binding to the promoter region of *ompW*, a non-radioactive EMSA was performed according to the protocol described by De la Cruz *et al.* (2007). The probes were obtained by PCR using specific primers pLacZ_OmpW_−600Fw and pLacZ_OmpW_+1Rv to amplify the promoter region of *ompW* (600 bp) and pOmpW_+1_Fw with pOmpW_+130_Rv (Table 2) for the negative control encompassing the coding region of *ompW* (130 bp). The PCR was performed under the following conditions: 10 min at 95 °C, followed by 30 cycles of 30 s at 95 °C, 30 s at 55 °C and 1 min at 72 °C, and a final extension of 10 min at 72 °C. PCR products used in EMSAs with mutations in the *mar*/*sox*/*rob* boxes A, B and C were generated using primers pLacZ_OmpW_-600Fw and pLacZ_OmpW_+1Rv, and plasmids pMutAB-*lacZ*, pMutAC-*lacZ*, pMutBC-*lacZ* and pMutABC-*lacZ* as templates. Both the promoter region and the negative control (~2 ng ml^−1^) were mixed with increasing amounts of purified MarA or SoxS in the presence of binding buffer [20 mM HEPES, 100 mM KCl, 2 mM MgCl_2_, 0.1 mM EDTA, 20 % (v/v) glycerol]. The mixture was incubated for 30 min at room temperature and loaded on a native 6 % polyacrylamide gel in 0.5× Tris/borate–EDTA buffer. The DNA bands were visualized by ethidium bromide staining on a UV transilluminator. All primers used in this work were designed using the Vector NTI 10 Software.

## Results

### *ompW* is positively regulated after menadione treatment

**Fig. 1.**
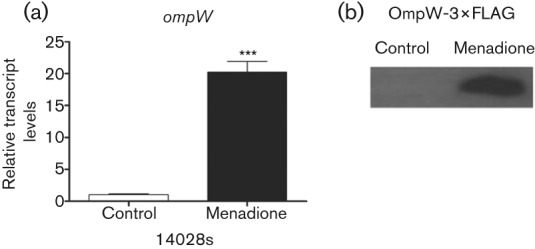
Effect of menadione on OmpW in *S. enterica* serovar Typhimurium** 14028s. Exponentially growing cells were exposed to menadione (50 µM) for 20 min. Controls received no treatment. (a) qRT-PCR was used to analyse *ompW* transcript levels from strain 14028s. Values are means±sd. Experiments were repeated three times and asterisks represent significant differences between control and treated cells (***P<0.001). (b) OmpW-3×FLAG protein was detected by Western blotting. Each lane was loaded with 10 µg total protein. Experiments were repeated three times and a representative result is shown.

In order to evaluate the effect of menadione on the expression of *ompW*, we analysed the transcript levels in a *S. enterica* serovar Typhimurium** wild-type strain treated with the toxic compound. As shown in Fig. 1(a), *ompW* transcript levels were increased (20.22±1.70 fold changes) as compared with control untreated cells. To correlate the changes in the levels of transcripts with the gene product OmpW (~21 kDa) and evaluate possible post-transcriptional regulation, we constructed translational fusions in the wild-type strain and evaluated their levels as described in Methods. In agreement with qRT-PCR analysis, OmpW levels were increased as compared with those of untreated cells (Fig. 1b).

### *ompW* is positively regulated by MarA and SoxS

**Fig. 2.**
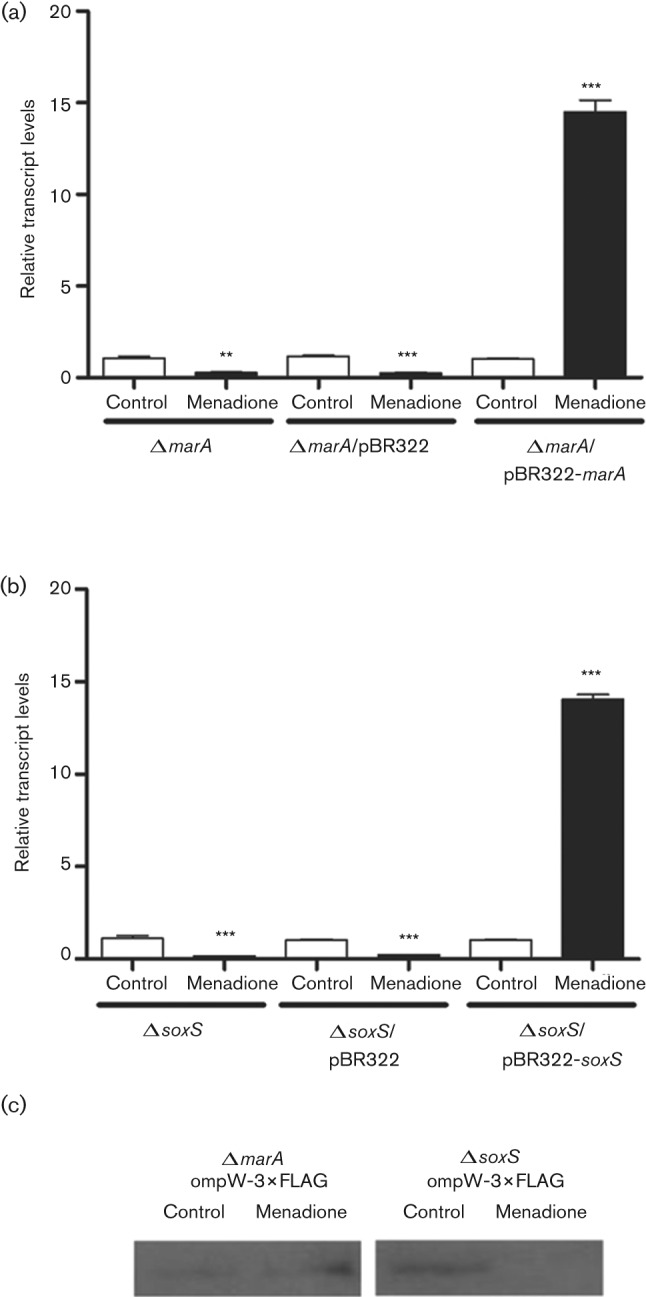
Effect of menadione on OmpW expression in *S. enterica* serovar Typhimurium** Δ*marA* and Δ*soxS* strains. Exponentially growing cells were exposed to menadione (50 µM) for 20 min. Controls received no treatment. *ompW* transcripts were detected by qRT-PCR in a *marA* mutant, Δ*marA*, genetically complemented strain Δ*marA*/pBR322-*marA* and a strain carrying the empty vector, Δ*marA*/pBR322 (a), and in a *soxS* mutant, Δ*soxS*, genetically complemented strain Δ*soxS*/pBR322-*soxS* and a strain carrying the empty vector, Δ*soxS*/pBR322 (b). Experiments were repeated three times and asterisks represent significant differences between control and treated cells for each strain. Values are means±sd (**P≤0.05, ***P≤0.001). (c) OmpW-3×FLAG protein was detected in a Δ*marA* : : FRT *ompW*-3×FLAG and Δ*soxS* : : FRT *ompW*-3×FLAG strain. Each lane was loaded with 10 µg total protein. Experiments were repeated three times and a representative result is shown.

We previously demonstrated that SoxS positively regulates *ompW* in response to paraquat (Gil *et al.*, 2009). Since MarA, SoxS and Rob co-regulate several genes (Martin & Rosner, 2002), we evaluated *ompW* transcript levels in the different genetic backgrounds by qRT-PCR after exposure to menadione. As shown in Fig. 2a, b, in strains Δ*marA* and Δ*soxS* the transcript levels were decreased after exposure to menadione (0.27±0.08 and 0.17±0.01 fold changes, respectively, versus a 20-fold increase in wild-type cells exposed to the toxic compound). In contrast, in the *rob* mutant strain they were upregulated to similar levels as those found in the wild-type strain after the treatment (11.42±0.52 fold change, Fig. S1a, available with the online version of this paper). As expected, *in trans* complementation of strains Δ*marA* and Δ*soxS* restored the positive regulation observed in the wild-type strain (Fig. 2a, b).

To further confirm our result, we generated translational fusions of *ompW* in the Δ*marA* and Δ*soxS* genetic backgrounds and determined the protein levels by performing a Western blot. As shown in Fig S1(a), the positive regulation observed in the wild-type strain after menadione treatment was retained in the Δ*rob* strain (Fig S1b), while in Δ*marA* and Δ*soxS* strains it was abolished (Fig. 2a–c). Taken together, qRT-PCR and Western blot analyses suggest that MarA and SoxS are required to positively regulate *ompW* in response to menadione, while Rob is not involved in this response.

### MarA and SoxS bind to the *ompW* promoter region

**Fig. 3.**
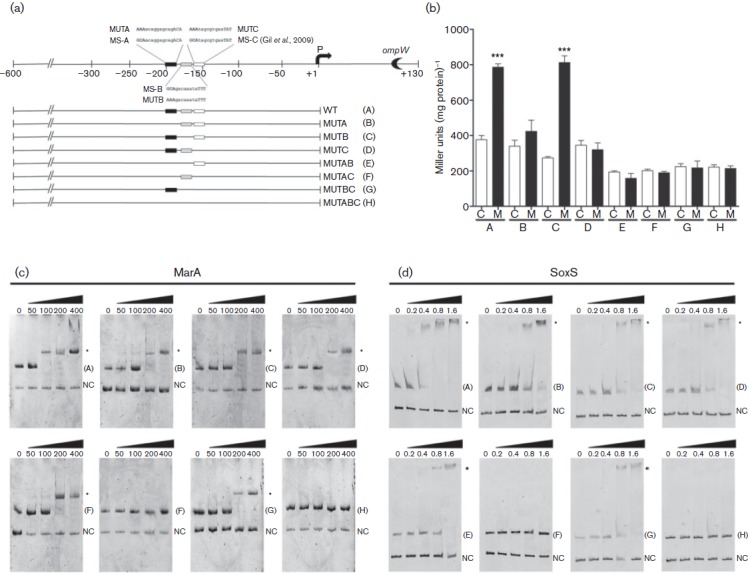
Evaluation of the binding of MarA and SoxS at the *ompW* promoter and functionality of the *marsox* boxes. (a) Schematic representation of *marsox* boxes MS-A (black), MS-B (grey) and MS-C (white), and substitutions generated at the *ompW* promoter (native and substituted bases are in upper case). Marsox boxes are represented by rectangles. The absence of a rectangle in the construct represents a mutation of the corresponding binding site. Letters A–H identify the constructs. (b) Expression of the wild-type and mutagenized regulatory region of *ompW* in *S. enterica* serovar Typhimurium. The constructs A–H are shown. Cells were grown to OD_600_ ~0.4 and treated with 50 µM menadione (M) for 20 min and β-galactosidase activity was measured. Controls (C) received no treatment. Values represent the means±sd of three independent experiments (*** *P*≤0.001). (c) and (d) EMSA using increasing concentrations of MarA (nM) or SoxS (µm), respectively, with fragments A–H indicated. NC, negative control. The interactions were resolved by native PAGE (6 %). Bands were visualized by ethidium bromide staining. Asterisks indicate DNA–protein interaction.

To evaluate if the regulation of *ompW* by MarA and SoxS was due to a direct interaction, we performed EMSAs to determine if the purified proteins were able to bind to its promoter region. The bioinformatic analysis predicted the presence of three putative *mar*/*sox*/*rob* boxes (Fig. 3a), two novel ones named MS-A and MS-B, in addition to the previously identified SoxS-binding site (MS-C) described by Gil *et al.* (2009). All three binding sites presented the two characteristic elements described at *marsox* boxes, CWA and the highly conserved GCAY (Li & Demple, 1994), which are required for the stability of the interaction and for protein binding, respectively (Li & Demple, 1996). To confirm the interactions, we performed EMSAs using a PCR product spanning the promoter region from positions −600 to +1 with respect to the transcription start site, with increasing concentrations of purified MarA or SoxS. As a negative control, a PCR product that included a region from +1 to +130 of *ompW* was used. Both MarA and SoxS were able to bind to the wild-type promoter (Fig. 3c, d), although at different concentrations. MarA generated a change in the electrophoretic mobility at a concentration of 100 nM, while SoxS required 400 nM (Fig. 3c, d, respectively, fragment A). Mutation of the GCAY element to AAAY (Fig. 3a) in the three predicted boxes required doubling of the amount of both MarA and SoxS to generate a shift in the electrophoretic mobility as compared with that of the wild-type promoter, while mutating MS-A and MS-C together completely abolished the interaction with both proteins (Fragments F and H, Fig. 3c, d), suggesting that they are required for binding *in vitro*.

### The promoter region of *ompW* has two functional *marsox* boxes

To determine which *marsox* boxes were functional *in vivo*, we constructed transcriptional fusions of the *ompW* promoter region with the fragments used for EMSAs, schematized in Fig. 3(a). The different constructs were transformed into strain 14028s and β-galactosidase activity was measured. All activities were compared with that of strain 14028s with the wild-type construct (A). Cells containing the wild-type promoter (A) or MS-B mutated (C) showed a twofold increase in β-galactosidase activity after exposure to the toxic compound (Fig. 3b), indicating that MS-B is dispensable for *ompW* upregulation by MarA and SoxS in response to menadione. However, individually mutating MS-A and MS-C or mutating both together resulted in no regulation after exposure to the toxic compound (Fig. 3b, fragments B, D, E, F, G and H), indicating that both sites are required for the positive regulation by MarA and SoxS in strain 14028s, results which are in agreement with those from EMSAs.

### Both MarA and SoxS are required for *ompW* positive regulation

**Fig. 4.**
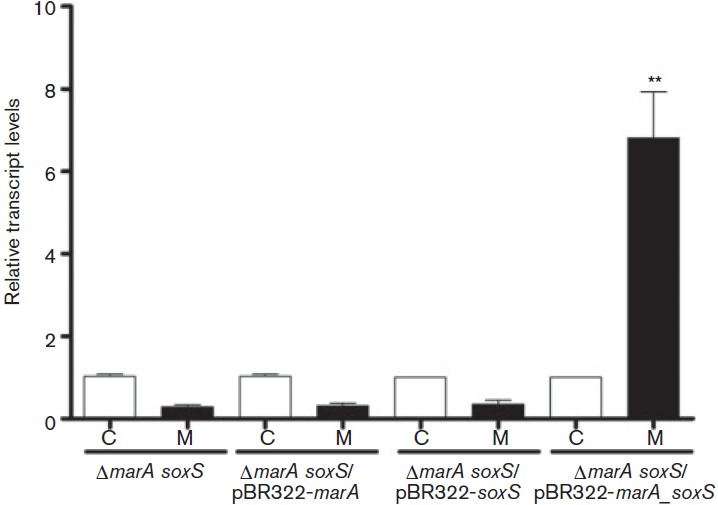
Requirement for MarA and SoxS for *ompW* positive regulation. *ompW* transcript levels were measured in a double *marA soxS* mutant strain, individually complemented (Δ*marA soxS*/pBR322-*marA* and Δ*marA soxS*/pBR322-*soxS*) and complemented with both genes (Δ*marA soxS*/pBR322-*marA*_*soxS*). Exponentially growing cells were exposed to menadione (50 µM) for 20 min. Controls received no treatment. Experiments were repeated three times and asterisks represent statistically significant differences between the control and treated cells for each strain (**P≤0.005); values are means±sd. C, control; M, menadione.

To determine whether MarA and SoxS individually regulated *ompW* in response to menadione or if they were both required, we generated a double Δ*marA soxS* strain and measured *ompW* transcript levels in the presence or absence of menadione. As observed in the individual mutants, *ompW* levels remained decreased in the Δ*marA soxS* strain after treatment with the toxic compound (0.29±0.05 fold change, Fig. 4). When the double mutant strain was complemented *in trans* with a plasmid carrying the *S. enterica* serovar Typhimurium *soxS* or *marA* gene (Δ*marA soxS*/pB322-*soxS* and Δ*marA soxS*/pB322-*marA*, respectively), the transcript levels remained decreased after menadione treatment (0.354±0.1 and 0.314±0.06 fold change, respectively, Fig. 4). In contrast, when the double mutant strain was complemented with a plasmid coding for both *marA* and *soxS* (Δ*marA soxS*/pB322-*marA*_*soxS*), the positive regulation was partially restored to levels similar to those observed in the wild-type strain exposed to menadione (6.81±1.13 fold change, Fig. 4), indicating that both MarA and SoxS are required for positive regulation.

### MarA and SoxS work cooperatively

**Fig. 5.**
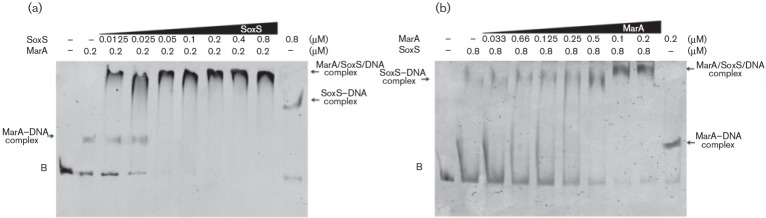
Interaction of MarA and SoxS at the *ompW* promoter region. Constant concentrations of purified MarA (a) or SoxS (b) were incubated with increasing concentrations of SoxS or MarA, respectively, and the wild-type promoter of *ompW* (fragment B). The interactions were resolved by native PAGE (6 %). Bands were visualized by ethidium bromide staining. The different DNA–protein complexes are indicated. −, No protein added.

Since both MarA and SoxS were required to positively regulate *ompW*, we hypothesized that the two proteins might act cooperatively. To evaluate this possibility, we performed EMSAs with the *ompW* promoter mutated at MS-B (fragment C, Fig. 3a), constant amounts of either MarA (Fig. 5a) or SoxS (Fig. 5b), and increasing amounts of the corresponding counterpart. When MarA remained constant (200 nM), adding increasing amounts of SoxS, from 0.0125 to 0.8 µM, resulted in a shift to a higher molecular mass than that generated by the individual proteins (Fig. 5a). Interestingly, even at the lower concentrations of SoxS (0.0125 µM) the high-molecular-mass complex was observed, while incubating with SoxS alone required 0.4 µM to produce a shift using the same DNA probe (Fig. 3b, fragment C), suggesting that the affinity of SoxS for the promoter region of *ompW* increases in the presence of MarA. In agreement with this, using a constant amount of SoxS (0.8 µM) and increasing amounts of MarA (33–200 nM) resulted in a similar shift with a higher molecular mass to that observed for the individual proteins (Fig. 5b). As observed for SoxS, lower levels of MarA were required to form the high-molecular-mass complex (100 nM) than when the protein was incubated alone with the *ompW* promoter (200 nM), suggesting the same increased affinity for the promoter region as observed in the case of SoxS. Taken together, our results indicate that both MarA and SoxS are required to positively regulate *ompW* and that they cooperatively bind to the promoter region.

## Discussion

The OmpW protein is an immunogenic 22 kDa (Jalajakumari & Manning, 1990) minor porin and has been related to osmoprotection (Hu *et al.*, 2005), the efflux and resistance towards paraquat (Gil *et al.*, 2009) and the influx of hydrogen peroxide and hypochlorous acid (Morales *et al.*, 2012). It is regulated by diverse environmental conditions including temperature, salinity, nutrient availability, oxygen levels (Nandi *et al.*, 2005), paraquat (Gil *et al.*, 2009) and reactive oxygen species (Morales *et al.*, 2012), among others, and is differentially regulated at the transcriptional level by FNR (anaerobiosis), ArcA (H_2_O_2_ and NaOCl) and SoxS (paraquat) (Bouchal *et al.*, 2010; Morales *et al.*, 2012; Gil *et al.*, 2009).

In this work, we demonstrate that both SoxS and MarA, whose response overlap and together co-regulate over 40 genes (Aono *et al.*, 1998; Martin *et al.*, 1999, 2000; Martin & Rosner, 2002), were required for the positive regulation of *ompW* after menadione treatment (Figs 2 and 4). Consistent with this, both transcription factors are upregulated in response to the toxic compound (Fig S2), and SoxS is required to regulate *ompW* in response to paraquat (Gil *et al.*, 2009). Our results indicate that there are two functional *mar*/*sox* boxes that are required for the positive regulation in response to menadione (Fig. 3). These sites are located approximately 100 nt upstream from the ArcA-binding site (from −70 to −55), required for the negative regulation in response to H_2_O_2_ and NaOCl (Morales *et al.*, 2012). This suggests that under the assayed conditions, ArcA is not active, since, as observed in the presence of NaOCl, when ArcA, MarA and SoxS are present, *ompW* is negatively regulated. It is plausible to speculate that under those conditions ArcA could bind to the −35 element and impede binding of the sigma factor, explaining why, although both MarA and SoxS are present, *ompW* is negatively regulated. In contrast, in response to menadione ArcA could be inactive, allowing MarA and SoxS to exert their regulation, although this has not been evaluated.

Our results indicate that Rob is not involved in the regulation of *ompW* (Fig. S1), indicating that it is regulated in a different manner as compared with other genes that are members of the extensively studied *mar*/*sox*/*rob* regulon, like *tolC* in *E*. *coli*, which is positively regulated by all of them in response to salicylate, paraquat and 2,2′-dipyridyl (Zhang *et al.*, 2008). In addition, previous work indicates that *rob* is repressed by MarA due to steric hindrance and in *E*. *coli* SoxS modulates its expression in response to paraquat (McMurry & Levy, 2010; Michán *et al.*, 2002). Furthermore, studies in *S. enterica* serovar Typhimurium** showed that the transcript and protein levels of *marA* and *soxS* are increased, while those of *rob* are lowered in a wild-type strain treated with sodium hypochlorite (Collao *et al.*, 2012), suggesting a similar mechanism.

To investigate the mechanism by which MarA and SoxS regulate *ompW*, we performed EMSAs and used transcriptional fusions of the promoter region (Figs 3 and 5). Our results indicate that both proteins are required for positive regulation and that they act in a cooperative manner (Figs 4 and 5). In this context, several reports provide evidence that two transcription factors work cooperatively in response to the same signal, as in *Vibrio vulnificus*, where the *nan* operon is negatively regulated by CRP and NanR in the presence of *N*-acetylmannosamine 6-phosphate (Kim *et al.*, 2011). Also, in *E. coli* CRP requires the presence of RhaR to efficiently activate *rhaSR in vivo* in response to l-rhamnose (Wickstrum *et al.*, 2005). Similarly, studies in *Haemophilus influenzae* suggest that CRP and SiaR regulate their respective operators by simultaneously binding to an intergenic region between *nan* and *siaPT*, where SiaR functions as both a repressor and activator, using glucosamine-6-phosphate as a co-activator, and interacts with CRP to regulate these divergent promoters (Johnston *et al.*, 2010). Furthermore, in *Myxococcus xanthus* MrPC2 and FruA bind cooperatively to three sites at the *fmgE* promoter region, and it has been proposed that one site is necessary to recruit MrpC2 and FruA to the promoter, while the other two are required to activate it (Son *et al.*, 2011). However, most of these studies mainly show that the effect on the target genes is synergic. In contrast, our results indicate that MarA and SoxS are required to positively modulate *ompW* expression (Figs 4 and 5). To our knowledge, this is the first report demonstrating such an effect. Further studies addressing whether this is a common feature of regulation of gene expression by MarA and SoxS, novel targets subject to similar regulation, and the mechanism by which these proteins interact are under examination in our laboratory.
